# Homeostatic normalization of alpha brain rhythms within the default-mode network and reduced symptoms in post-traumatic stress disorder following a randomized controlled trial of electroencephalogram neurofeedback

**DOI:** 10.1093/braincomms/fcad068

**Published:** 2023-03-16

**Authors:** Andrew A Nicholson, Maria Densmore, Paul A Frewen, Richard W J Neufeld, Jean Théberge, Rakesh Jetly, Ruth A Lanius, Tomas Ros

**Affiliations:** School of Psychology, University of Ottawa, Ottawa, Canada; Atlas Institute for Veterans and Families, Royal Ottawa Hospital, Ottawa, Canada; The University of Ottawa’s Institute of Mental Health Research, Royal Ottawa Hospital, Ottawa, Canada; Department of Medical Biophysics, Western University, London, Canada; Department of Neuroscience, Western University, London, Canada; Imaging, Lawson Health Research Institute, London, Canada; Department of Neuroscience, Western University, London, Canada; Department of Psychology, Western University, London, Canada; Department of Neuroscience, Western University, London, Canada; Department of Psychiatry, Western University, London, Canada; Department of Psychology, Western University, London, Canada; Department of Psychiatry, Western University, London, Canada; Department of Psychology, Western University, London, Canada; Department of Medical Biophysics, Western University, London, Canada; Imaging, Lawson Health Research Institute, London, Canada; Department of Diagnostic Imaging, St. Joseph’s Healthcare, London, Canada; Defence Research and Development Canada, Toronto, Canada; Department of Neuroscience, Western University, London, Canada; Department of Psychiatry, Western University, London, Canada; Imaging, Lawson Health Research Institute, London, Canada; Departments of Neuroscience and Psychiatry, University of Geneva; Campus Biotech, Geneva, Switzerland; Centre for Biomedical Imaging (CIBM), Geneva, Switzerland

**Keywords:** post-traumatic stress disorder (PTSD), neurofeedback, EEG, alpha rebound

## Abstract

Collective research has identified a key electroencephalogram signature in patients with post-traumatic stress disorder, consisting of abnormally reduced alpha (8–12 Hz) rhythms. We conducted a 20-session, double-blind, randomized controlled trial of alpha desynchronizing neurofeedback in patients with post-traumatic stress disorder over 20 weeks. Our objective was to provide mechanistic evidence underlying potential clinical improvements by examining changes in aberrant post-traumatic stress disorder brain rhythms (namely, alpha oscillations) as a function of neurofeedback treatment. We randomly assigned participants with a primary diagnosis of post-traumatic stress disorder (*n* = 38) to either an experimental group (*n* = 20) or a sham-control group (*n* = 18). A multichannel electroencephalogram cap was used to record whole-scalp resting-state activity pre- and post-neurofeedback treatment, for both the experimental and sham-control post-traumatic stress disorder groups. We first observed significantly reduced relative alpha source power at baseline in patients with post-traumatic stress disorder as compared to an age/sex-matched group of neurotypical healthy controls (*n* = 32), primarily within regions of the anterior default mode network. Post-treatment, we found that only post-traumatic stress disorder patients in the experimental neurofeedback group demonstrated significant alpha resynchronization within areas that displayed abnormally low alpha power at baseline. In parallel, we observed significantly decreased post-traumatic stress disorder severity scores in the experimental neurofeedback group only, when comparing baseline to post-treatment (Cohen’s *d* = 0.77) and three-month follow-up scores (Cohen’s *d* = 0.75), with a remission rate of 60.0% at the three-month follow-up. Overall, our results indicate that neurofeedback training can rescue pathologically reduced alpha rhythmicity, a functional biomarker that has repeatedly been linked to symptoms of hyperarousal and cortical disinhibition in post-traumatic stress disorder. This randomized controlled trial provides long-term evidence suggesting that the ‘alpha rebound effect’ (i.e. homeostatic alpha resynchronization) occurs within key regions of the default mode network previously implicated in post-traumatic stress disorder.

## Teaser

Nicholson *et al.* report findings from a double-blind randomized controlled trial investigating changes in EEG-based measures following alpha-rhythm neurofeedback training in post-traumatic stress disorder. They found that only the experimental group demonstrated alpha resynchronization after treatment (alpha rebound) and reduced symptoms.

## Graphical Abstract

**Graphical abstract:**
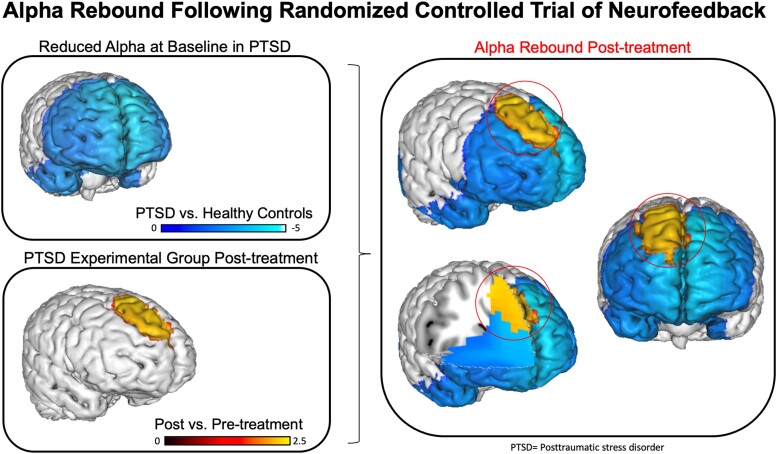

## Introduction

Traumatic experiences can often result in the development of post-traumatic stress disorder (PTSD),^1^ a psychiatric illness that involves symptoms of persistent intrusive recollections, avoidance of trauma-related stimuli, negative alterations in cognitions and mood and marked alterations in arousal and reactivity.^1^ Despite the high prevalence rate of PTSD worldwide and its debilitating psychopathology,^2-5^ up to 40% of patients with PTSD can fail to respond to frontline treatments such as psychotherapy or pharmacotherapy.^6-8^ It is therefore critical to develop novel, neuroscientifically-guided treatments that target more directly the neural mechanisms implicated in PTSD.^9-13^ Indeed, research investigating the treatment efficacy of neurofeedback (NFB), a non-invasive brain-computer interface that can directly regulate aberrant neural dynamics tied to psychopathology, suggests that this treatment may represent a promising new avenue toward recovery from PTSD.^11,14-21^

### Neurofeedback studies in PTSD

Recent studies and systematic reviews in the field of NFB suggest that this intervention is associated with significant symptom improvements among individuals with PTSD^22-25^ and that NFB may be particularly beneficial among individuals who have been resistant to standard treatments.^19,21,26^ With regard to neural targets for NFB treatment, several studies suggest covariation between EEG alpha rhythms (8–12 Hz) and intrinsic connectivity networks^27,28^ that are implicated in PTSD psychopathology.^10,26^ Simultaneous EEG-fMRI studies have found that alpha fluctuations are positively correlated with activity within the default mode network (DMN),^29-31^ a network implicated in self-referential processing^32^ and autonomic arousal.^33-35^ Among persons with PTSD, alpha-rhythm reductions have been associated with PTSD symptoms,^31,36^ particularly those of chronic hyperarousal.^26,31,37-41^ This is consistent with classical studies indicating that alpha rhythms (8–12 Hz) predominate during states of wakeful ‘rest’ associated with increased cortical inhibition i.e. reduced excitatory/inhibitory (E/I) balance.^42-44^ Conversely, alpha rhythms significantly attenuate during states of high behavioural arousal.^45^ In PTSD, resting-state alpha-rhythm reductions have been frequently observed within the main hubs of the DMN, including the medial prefrontal cortex (mPFC) and posterior cingulate cortex (PCC).^31^ Importantly, several studies have provided preliminary evidence suggesting that alpha-based NFB may be clinically effective for both reducing PTSD symptoms and normalizing aberrant neural dynamics associated with the disorder.^11,18,24,46,47^

Indeed, single-session mechanistic studies of EEG-NFB among individuals with PTSD have previously shown that one treatment session of alpha-based NFB resulted in hyperarousal symptom decreases.^18,47^ This was also associated with plastic changes in functional connectivity within the DMN and salience network (SN),^18,47^ where functional alterations within the DMN and SN have previously been shown to be highly implicated in PTSD and its successful treatment.^9,48-51^ Furthermore, a single-session of alpha-rhythm NFB was also found to induce a shift in amygdala complex functional connectivity away from the hippocampus and defence processing areas in the midbrain (periaqueductal grey) towards vmPFC areas involved in executive functioning and emotion regulation.^52^ Our group has also shown that one treatment session of alpha-reducing NFB can restore (increase) abnormally attenuated alpha rhythms in PTSD patients towards levels found in neurotypical healthy controls post-intervention.^47^ This counterintuitive shift towards normalization of alpha rhythms post-intervention, referred to as the ‘alpha rebound effect’ has been proposed to occur via homeostatic plasticity mechanisms that regulate E/I balance in the cortex.^18,41,47^ Importantly, this alpha rebound effect, or resynchronization following alpha desynchronizing NFB, has been recently replicated in an independent multi-session study among individuals with PTSD from Rwanda and was found to be associated with clinically relevant symptom reductions.^46^

In support of this, several multi-session randomized controlled trials (RCTs) of EEG-NFB have also been conducted in PTSD, and together, suggest high treatment efficacy when addressing treatment-resistant PTSD.^19,21,24,53^ Indeed, we previously conducted a 20-session alpha desynchronizing EEG-NFB RCT in PTSD and found that significantly reduced PTSD severity scores in the experimental group were associated with a shift towards normalization of DMN and SN connectivity patterns.^11^ Elsewhere, an RCT in patients with chronic PTSD found that 24 sessions of EEG-NFB (to decrease delta/theta/high beta, and increase alpha) led to significant improvements in both PTSD symptoms and patients’ capacity for emotion regulation.^19^ Importantly, participants in this study consisted of repeatedly traumatized individuals who had not responded to at least six months of trauma-focused psychotherapy.^19^ Similarly, a recent 24-session EEG-NFB RCT in children with severe developmental trauma (to decrease delta/theta/high beta, and increase the posterior resting rhythm) demonstrated significant reductions in PTSD symptoms and improved executive functioning.^21^ Furthermore, a 16-session RCT of alpha-based NFB (involving alpha upregulation) over eight weeks demonstrated significantly reduced PTSD symptoms which persisted one month after treatment, where NFB was also associated with reduced symptoms of anxiety, depression and insomnia.^53^ Critically, however, RCTs of EEG-NFB have yet to examine treatment-associated improvements in aberrant PTSD brain rhythms post-intervention. More specifically, observations of an ‘alpha rebound effect’ following alpha desynchronizing EEG-NFB have yet to be replicated in a double-blind, RCT in PTSD.

### Study objective and hypotheses

For the first time, we present findings from a double-blind, randomized, sham-controlled trial investigating changes in EEG-based measures following alpha-rhythm NFB training over a 20-week period. Our central goal was to provide mechanistic evidence underlying clinical improvements by examining changes in relative alpha power over the course of neurofeedback treatment.

We first compared baseline relative alpha source power between all PTSD patients and neurotypical healthy controls in order to better characterize aberrant neural dynamics related to psychopathology before NFB. In line with the extant literature,^31,36,47^ we hypothesized that as compared to controls, individuals with PTSD would demonstrate reduced alpha power at baseline. Second, based on previous exploratory studies,^18,46^ we hypothesized that NFB training would result in a normalization (increase) of relative alpha power within cortical areas that were found to be disrupted at baseline, consistent with mechanisms of homeostatic neuroplasticity and the alpha rebound effect.^47^ Finally, we predicted that the PTSD experimental group would demonstrate significant reductions on the primary outcome measure of PTSD severity as compared to the sham-control group.

## Materials and methods

### Participants

Our sample consisted of 72 participants (*n* = 40 PTSD, *n* = 32 neurotypical healthy controls, see Table 1). Individuals who met criteria for a primary diagnosis of PTSD were randomized to either the experimental EEG-NFB group or the sham-control EEG-NFB group. A total of two participants were excluded from the analysis as a result of missing data, and not falling within the inclusion/exclusion criteria for the current study (i.e. meeting diagnostic criteria for bipolar disorder and substance use disorder post-NFB), resulting in a final sample size of *n* = 20 participants with PTSD in the experimental EEG-NFB group, and *n* = 18 participants with PTSD in the sham-control EEG-NFB group. The neurotypical healthy control group was utilized to compare baseline EEG signatures before the intervention, and this group did not receive NFB treatment.

**Table 1:** Participant demographic and clinical information

	PTSD experimental group	PTSD sham-control group	Neurotypical healthy control group
N	20	18	32
Sex	13 females	14 females	22 females
Age	39.20 (12.08)	46.28 (12.37)	42.40 (10.7)
CAPS-Total	36.52 (9.71)	39.94 (7.83)	
CTQ	54.50 (20.96)	63.88 (19.94)	
MDI-Total	52.15 (14.32)	67.88 (20.79)	
MDD	current = 6, past = 9	current = 7, past = 5	
Somatization disorder	current = 1, past = 0	current = 3, past = 0	
Specific phobia	current = 0, past = 0	current = 1, past = 0	
Medication	12	12	

Brackets indicate standard deviation. For comorbid diagnoses, c = current and p = past diagnoses. PTSD groups did not differ with regard to CAPS, CTQ, MDI scores and psychiatry comorbidities. CAPS (normalized to CAPS-5), CTQ (*none or minimal childhood trauma = 25–36, moderate = 56–68, extreme trauma > 72*).

There were no statistically significant differences between groups with respect to age and biological sex (see Table 1). Prevalence of current psychiatric comorbidities did not differ significantly between the PTSD experimental and sham-control groups (experimental group: major depressive disorder (MDD) *n* = 6, somatization disorder *n* = 1; sham-control group: MDD *n* = 7, somatization disorder *n* = 3, specific phobia *n* = 1). Regarding criterion A trauma exposure, PTSD diagnoses in the experimental NFB group were associated with military occupational trauma (*n* = 4), first responder occupational trauma (*n* = 2) and civilian physical/sexual abuse or neglect (*n* = 14). Similarly, PTSD diagnoses in the sham-control group were associated with military occupational trauma (*n* = 3), first responder occupational trauma (*n* = 1) and civilian physical/sexual abuse or neglect (*n* = 14). Critically, trauma type did not differ significantly between groups.

Participants with PTSD were recruited from 2014 to 2018 through referrals from healthcare professionals, psychology and psychiatric clinics, community programs for traumatic stress and posters/advertisements within the community. Inclusion criteria for the PTSD groups included a primary diagnosis of PTSD as determined using the clinician-administered PTSD scale [CAPS; versions IV (*n* = 4) and 5 (*n* = 34)] and the structured clinical interview for DSM-IV (SCID).^54-56^ Exclusion criteria for PTSD patients included: (i) alcohol dependency or substance use disorder not in sustained full remission within the last three months; (ii) lifetime diagnosis of bipolar or psychotic disorders; (iii) active participation in another primary trauma-focused psychotherapy; (iv) past or current biofeedback treatment; (v) acute suicidality within the past three months; (vi) self-injurious behaviours in the past three months requiring medical attention; and (vii) unstable living conditions or current involvement in a violent relationship.

The healthy control group consisted of *n* = 32 neurotypical adult participants matched for age/sex from the Human Brain Institute normative database (http://www.hbimed.com/). Here, inclusion/exclusion criteria were: (i) an uneventful perinatal period; (ii) no previous history of head injury with cerebral symptoms; (iii) no history of neurological or psychiatric disorders; (iv) no current medication or drug use; (v) no convulsions; and (vi) normal mental and physical development. All participants’ EEG recordings were performed using the same EEG amplifier (Mitsar-201) and behavioural condition (eyes closed). Notably, these were subjects who participated in a database building project which focused on collecting EEG reference data from neurotypical healthy controls. The project was sponsored by the Brain and Trauma foundation from Chur, Switzerland.

The number of individuals with PTSD currently receiving psychotropic medications (*n* = 24) did not differ significantly between the experimental group (*n* = 12) and the sham-control groups (*n* = 12), nor did the type of medications, which included antidepressants (*n* = 19), atypical antipsychotics (*n* = 6), sedatives (*n* = 8) and stimulants (*n* = 2). Additionally, participants with PTSD who were taking psychotropic medications were on a stable dose prior to the start of the NFB trial and were asked not to alter their medication regime.

This study was approved by the Research Ethics Board (REB) at Western University, Canada; participants gave written informed consent and received financial compensation. Experimental hypotheses were not indicated within participant facing study documents (i.e. the letter of information and informed consent documents). Participants meeting inclusion criteria for the PTSD groups were informed that they would be randomly assigned to either the experimental or sham-control NFB arms, and that we aimed to examine whether they could learn to therapeutically control neural signals. Participants in the sham-control neurofeedback group were offered active EEG-NFB following the three-month follow-up assessment. This study was not pre-registered as a clinical trial; hence, we were highly restrictive with the outcome measures we examined, with the primary outcome measure being PTSD severity scores (i.e. CAPS).

### Experimental procedures

As reported elsewhere,^11^ baseline assessments were conducted using the CAPS, SCID, childhood trauma questionnaire (CTQ)^57^ and the multiscale dissociation inventory (MDI).^58^ Subsequently, all participants underwent a baseline resting-state functional magnetic resonance imaging (fMRI) scan (fMRI results reported in separate manuscript^11^). One week later, participants with PTSD then began weekly sessions dedicated to EEG-NFB over a 20-week period (targeting alpha downregulation for 20 min) with pre- and post-NFB resting-state EEG recordings. We conducted post-treatment assessments using the CAPS and SCID one week after the last EEG-NFB session. During this visit, we also collected post-treatment resting-state fMRI scans.^11^ Additionally, we conducted follow-up assessments using the CAPS and SCID three months after the last EEG-NFB session. Double-blinding was maintained throughout the entire study.

### Clinical and demographic data analyses

Using SPSS v26, we first compared PTSD experimental and sham-control group baseline values on the CAPS, CTQ and MDI, in addition to participant age, using independent sample *t*-tests. We also compared biological sex and current psychiatric comorbidities (which included MDD, somatization disorder and specific phobias) between the experimental and sham-control groups using Pearson’s chi-squared and Fisher’s exact tests. Baseline clinical comparisons were Bonferroni corrected for multiple comparisons (*P* = 0.05/3). Group comparisons on age and biological sex were similarly conducted between the neurotypical healthy control group and the collective PTSD group.

The primary outcome measure of the current EEG-NFB trial was a change in PTSD severity scores as evaluated by the CAPS. In order to compare CAPS-5 and earlier CAPS-IV assessments, we first normalized all scores to the CAPS-5 scale. Specifically, we divided participant’s CAPS-IV scores (four participants in total, utilized prior to the release of CAPS-5) with the maximum score for the CAPS-IV and multiplied this by the maximum score for the CAPS-5.^11^ In order to examine NFB-associated changes on CAPS, we conducted a split plot repeated measures ANOVA with the between-subjects factor of group (experimental and sham groups) and the within-subjects factor of time (pre-NFB, post-NFB, three-month follow-up). Paired-sample *t*-tests were then used to examine within group changes on the CAPS. Independent sample *t*-tests were also used to compare CAPS scores between PTSD groups at post-NFB and at the three-month follow-up. *Post hoc* tests were Bonferroni corrected for multiple comparisons (*P* = 0.05/6) and effect sizes were estimated using Cohen’s *d* (*dz*). Homogeneity of variance and normality assumptions remained intact for all analyses.

### EEG neurofeedback paradigm

**Figure 1:**
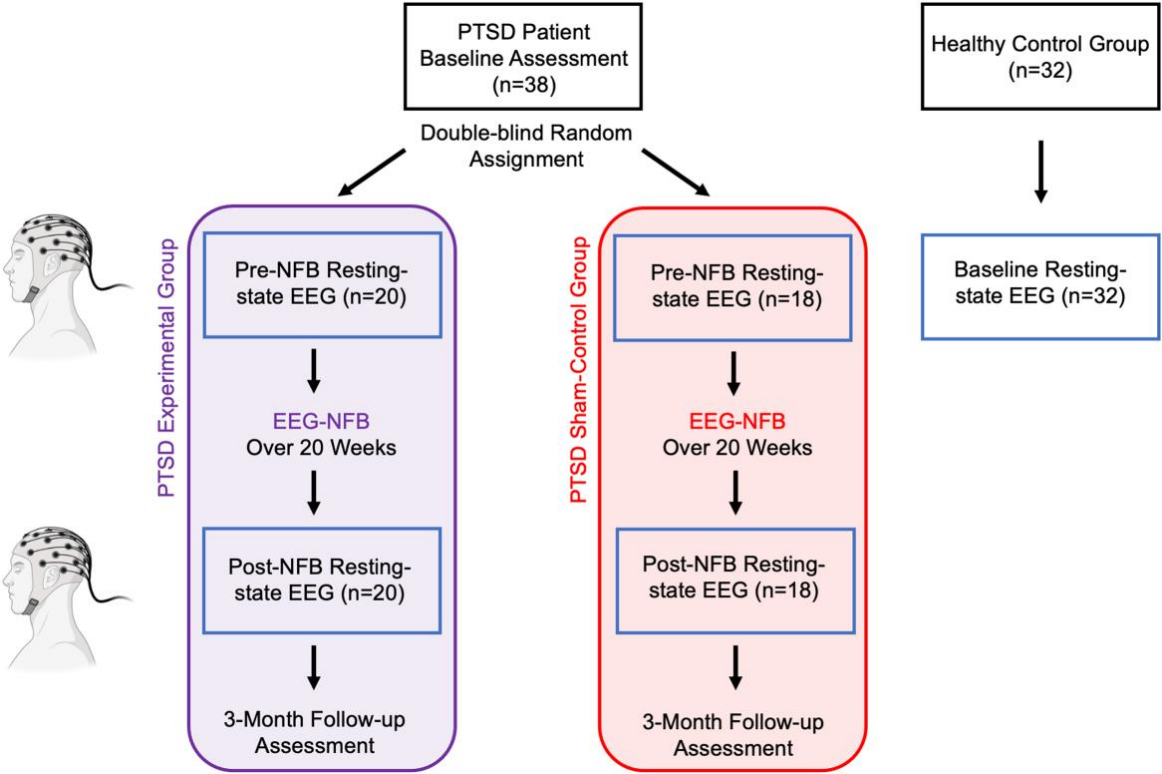
**Neurofeedback experimental design with pre- and post-intervention resting-state EEG recordings.** All PTSD patients were compared to neurotypical healthy controls at baseline on resting-state EEG recordings. PTSD patients were randomly allocated (double-blind) to either the active experimental group or sham-control group. Pre- versus post-intervention changes in resting-state EEG dynamics were compared between the experimental and sham-control groups. Clinical information was collected from the PTSD groups at baseline, post-intervention and at three-month follow-up.

Individuals with PTSD were randomly assigned to either the experimental or sham-control NFB groups under double-blind conditions (Fig. 1). Participants were required to both schedule and complete a minimum of 15 weekly EEG-NFB training sessions, with a maximum of 20 weekly sessions total being available [mean number of total completed sessions for experimental NFB group: 19.7 (SD 0.93); sham-control NFB group: 19.9 (SD 0.24)]. Here, no participant completed less than 17 total sessions and groups did not differ in the duration of treatment [average duration for the experimental group: 166.0 days (SD = 41.6); sham-control NFB group: 182.2 (SD = 39.7)]. We implemented the same EEG-NFB training protocol as described previously.^11,17,18^ During the first session, participants established goals for treatment and received psychoeducation consisting of an introduction to NFB technology. Specifically, we introduced to participants the idea that within our brains there are cells (neurons) that generate electrical activity at different frequencies, also known as ‘brain waves.’ We explained that we were interested in whether or not regulating certain brain waves might be a helpful approach in treating PTSD. We further communicated that NFB is a non-invasive tool by which we can measure brain waves in real-time in order to allow individuals to learn to regulate the feedback signal which may be associated with their symptoms. Additionally, during this visit, three-minute resting-state multichannel EEG recordings were collected, where participants were asked to relax under eyes closed conditions. Here, EEG signals were amplified with the Mitsar 21-channel EEG system (Mitsar-201, CE0537, Mitsar, Ltd) and all impedances were kept under 5 kΩ. In parallel, a bridged Pz channel with Ten20 electrolytic-paste was specifically used for NFB and was connected to a Phoenix A202 amplifier interfacing with EEGer 4.2 neurofeedback software (EEG Spectrum Systems) with right and left earlobes as ground and reference electrodes, respectively. The same multi-channel EEG/single-channel NFB recording was collected during the last NFB training session. During the second NFB session, 20 min of alpha-rhythm EEG-NFB training commenced, where participants could complete a maximum of 19 EEG-NFB training sessions in total. Individuals with PTSD in the experimental NFB group decreased alpha amplitude (8–12 Hz) using real-time EEG feedback signals from the midline parietal cortex (Pz-electrode). Conversely, PTSD patients in the sham-control NFB group received yoked sham-NFB signal, corresponding to a replayed feedback signal from a successful participant in the experimental group in order to ensure similar motivational states.^59^ The yoked-NFB signal replayed to participants in the sham-control group was matched on session number. Both research participants and the clinically trained research assistant delivering the NFB protocol were blind to group allocation and to how we generated the sham-NFB signal. Following randomization, a different, unblinded research assistant collected the data from a successful participant in the experimental group, and constructed the sham-NFB signal. This unblinded research assistant was also responsible for selecting the corresponding treatment arm on the NFB technology before the session with the participant began. Prior to all NFB training sessions, baseline three-minute resting-state EEG recordings without feedback were collected from the same Pz-electrode, in order to estimate the reward threshold for training (eyes open protocol). Importantly, EEGer sham-training gives the illusion of real NFB training, where signal feedback is still sensitive to real-time artefacts such as eye blinks and muscular activity. Participants did not receive explicit strategies on how to down-regulate the alpha signal during this trial and were told to explore individual strategies.

Similar to our previous investigations,^11,17,18^ the NFB signal was generated from the Pz-electrode as alpha rhythms are commonly maximal in this location.^60^ Participants completed EEG-NFB through interactive gaming. Here, two visual NFB interface options (i.e. options of visual presentation of the feedback signal) were provided to participants in order to be consistent with a trauma-informed model of treatment, take into account personal preference, and keep attention high over the 20-week trial. Participants could select continuous visual feedback in the form of either (i) a photo that had been divided into a grid, with individual grid pieces appearing as alpha amplitude was suppressed; or (ii) a cartoon character that moved across the screen as alpha amplitude was suppressed. Participants also received auditory feedback, in which a series of beeps occurred more frequently as they were suppressing alpha. Participants were informed that this auditory form of feedback was designed to complement the visual neurofeedback they were receiving.

The EEG signal used for online NFB was infinite impulse response band-pass filtered in order to extract alpha oscillations with an epoch size of 0.5 s and using a sliding window with 20% overlap (i.e. 100 ms). Here, the reward threshold was calibrated such that individuals would receive positive feedback ∼65% of the time and negative feedback ∼35% of the time. Reward thresholds were adjusted to meet the aforementioned ratio when participants received either disproportionately higher (>90%) or lower (<50%) rates of reward during NFB training. Reward threshold readjustment was made when positive feedback exceeded 90% (or went below 50%) based on the 30 s sliding window of mean alpha amplitude. Each 20-minute neurofeedback session was divided into seven training periods (6 × 3-minute time periods, and 1 × 2-minute time period). Adjustments were made at the beginning of training periods based on the EEG signal of the preceding 30 s.^18^ After each training period, simple bar graphs were displayed which represented the points earned (i.e. how much reward participants obtained) for each round.

### EEG neurofeedback recording and analysis

As reported elsewhere,^11^ scalp voltages were recorded using the Phoenix A202 2-channel EEG amplifier. The ground electrode was placed on the right earlobe and the reference electrode on the left earlobe. The EEG was recorded continuously, digitized at a sampling rate of 250 Hz, and then stored on a hard drive for offline analysis using a 0.5–40 Hz bandpass filter.

The raw EEG signal from the Pz-electrode was imported into the MATLAB toolbox EEGLAB, and statistically defined artefacting was then carried out with the FASTER plug-in,^61^ removing 1 s segments based on extremal deviations of amplitude and variance from the mean (−2 < *Z*-score > 2). Absolute alpha amplitude (8–13 Hz) was then estimated with a standard FFT approach using Welch’s method (MATLAB ‘pwelch’ function) and a Hanning windowing function (2 s epoch, 50% overlap). Given that some patients did not complete the full number of NFB training sessions, we used the expectation-maximization algorithm implemented in SPSS to replace the missing values *across-sessions*. The subsequent Group × Session ANOVA was then performed using these imputed values. For the case of missing values when calculating *within-session* statistics (i.e. between periods), we used the mean within-subject value across all sessions for that period.

### Multichannel EEG resting-state recordings and preprocessing

A multichannel EEG cap was used to measure whole-scalp activity during each resting-state recording, pre- and post-NFB treatment. This consisted of three-minute resting-state EEG recordings where participants were asked to relax under eyes closed conditions, during which participants were sitting against a headrest. Scalp voltages were recorded using a 19 Ag/AgCl electrode cap (Electro-cap International, Inc. www.electro-cap.com) according to the 10-20 international system. The ground electrode was placed equidistant between Fpz and Fz. Electrical signals were amplified with the Mitsar 21-channel EEG system (Mitsar-201, CE0537, Mitsar, Ltd. http://www.mitsar-medical.com) and all electrode impedances were kept under 5 kΩ. During recordings, electrodes were referenced to linked earlobes, and then the common average reference was calculated offline for further analysis. Resting-state EEG data were recorded at 250 Hz and bandpass filtered offline (0.5–40 Hz).

All EEG data were imported into the MATLAB toolbox EEGLAB v12 (http://sccn.ucsd.edu/eeglab/), where we subsequently used Infomax ICA decomposition to remove usual eye movements such as saccades or blinking.^62^ Recordings were further cleaned with an automated *Z*-score based method, using the FASTER plug-in,^61^ which rejected 1 s epochs that deviated from the mean by more than 2 SD.

### Offline source-space measures of resting-state EEG spectral power

Artefact-free, eyes closed, resting-state EEG data, were processed in MATLAB with the Brainstorm Toolbox (http://neuroimage.usc.edu/brainstorm/). In line with previous studies,^63^ we first computed a head model of the cortex surface for each EEG recording using the symmetric BEM from OpenMEEG^64^ and then estimated unconstrained cortical sources using the minimum-norm sLORETA algorithm implemented in Brainstorm. Here, we used the identity matrix as the noise covariance matrix. In order to normalize sources across participants, we projected (warped) the sources from each participant onto the MNI/ICBM152 template brain surface. Current source-density activities across all voxels were then band-pass filtered in the following four frequency bands: delta 1–4 Hz, theta 4–8 Hz, alpha 8–13 Hz and beta 14–30 Hz. For each participant, frequency bands were quantified in Brainstorm to examine differences in source power between the patient and control groups at baseline, as well as pre-to-post NFB.

*Spectral power.* Band-limited EEG power for each participant was estimated with a standard FFT approach using Welch’s method and a Hanning windowing function (2 s epoch, 50% overlap). For all group comparisons, we used the normalized measure of relative spectral power (i.e. % power), calculated as the ratio of the mean power in a specific EEG band and the broadband power (1–40 Hz).

### Offline EEG statistical analyses

For comparisons of EEG spectral power, we used the statistical pipelines directly available in the Brainstorm toolbox. We used nonparametric permutation testing^65^ to evaluate the statistical significance of voxel-wise spectral power difference between/within the experimental and sham-control groups. Specifically, permutation tests were performed across subjects for random effects inference. Under the null hypothesis of no spectral power difference in the source data between the two conditions, the labels between conditions A and B for each subject were randomly permuted, and the resulting data were used to compute a permutation *t*-statistic. Repeating this permutation procedure 10 000 times using Monte Carlo random sampling enabled us to estimate the empirical distribution of the *t*-statistic at each voxel and frequency thereby converting the original data into *P*-value thresholded statistical maps. All permutation tests were conducted using a two-tailed significance threshold of *P* < 0.05 corrected for multiple comparisons. Multiple comparison correction across frequencies was performed using the false discovery rate (FDR) procedure in the Brainstorm toolbox.^66^ Finally, we performed linear regression analyses to examine potential associations between changes in the CAPS (post-minus-pre NFB) and relative alpha power (post-minus-pre NFB). These analyses were conducted within SPM12 software using the ImCalc function which calculated post-minus-pre NFB *Z*-score change maps for each participant, which served as the dependent variables in linear regression analyses. We conducted these analyses separately for both the PTSD experimental and sham-control groups, using the same conservative FDR correction that was applied in the previous analyses.

## Results

### Demographic and clinical comparisons

We found that PTSD patients in the experimental and sham-control groups did not differ significantly at baseline with regard to global PTSD severity scores (CAPS total scores), exposure to childhood trauma as measured by the CTQ, and trauma-related dissociation scores as measured by the MDI (Table 1). These groups also did not differ significantly with regard to age, sex, or current psychiatric comorbidities (major depressive disorder, somatization disorder and specific phobias; see Table 1). The neurotypical control group was age and sex matched to the PTSD group.

### Neurofeedback improvements on PTSD symptom severity

**Figure 2:**
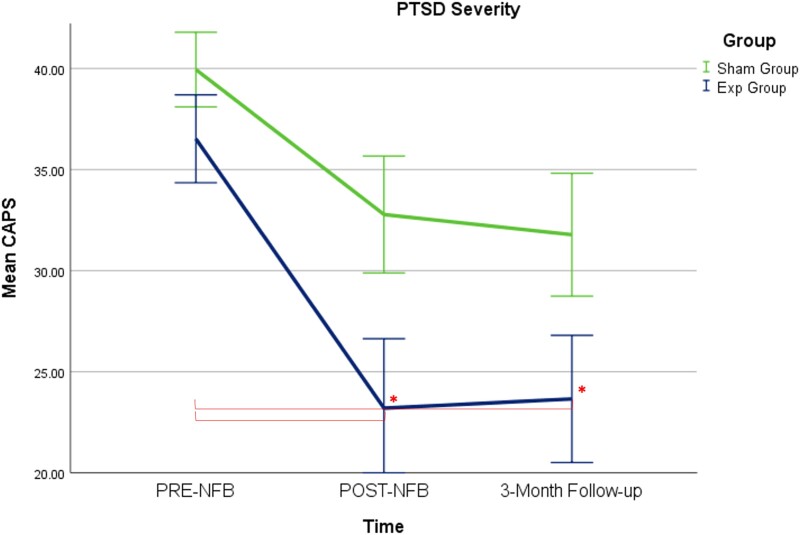
**Treatment induced changes in PTSD symptoms.** The primary outcome measure of PTSD severity (CAPS total scores) changed significantly over the NFB intervention for the experimental NFB group only as compared to baseline measures. Asterisks signify statistical significance for the paired-sample *t*-tests, corrected for multiple comparisons (*P* < 0.008). Acronyms: Exp = experimental neurofeedback PTSD group, Sham = sham-control PTSD group.

When examining changes in PTSD severity scores as measured by the CAPS, we found a significant main effect of time (*F*(2,70; Greenhouse–Geisser adjustment 1.43, 49.98) = 14.33, *η*_p_^2^ = 0.291, *P* < 0.0001), where the Group × Time interaction did not reach statistical significance (*F*(2,70; Greenhouse–Geisser adjustment 1.43, 49.98) = 0.834, *η*_p_^2^ = 0.023, *ns*) (Greenhouse–Geisser corrected). Nevertheless, *post hoc t*-tests revealed that only the PTSD experimental group demonstrated significant reductions on CAPS-totals scores from pre- to post-NFB (*t*(19) = 3.42, *P* = 0.003, *dz* = 0.77) and from pre-NFB to the three-month follow-up (*t*(18) = 3.26, *P* = 0.004, *dz* = 0.75) (see Table 2 and Fig. 2). Comparisons to baseline scores were found to be non-significant for the sham-control PTSD group from pre- to post-NFB (*t*(17) = 2.38, *ns*, *dz* = 0.56) and from pre-NFB to the three-month follow-up (*t*(17) = 2.68, *ns*, *dz* = 0.63). Finally, independent-samples *t*-tests comparing the experimental and sham-control PTSD groups at post-NFB and at the three-month follow-up did not reach statistical significance.

**Table 2:** Primary outcome measure PTSD severit*y*

	PTSD experimental group			PTSD sham-control group		
	Pre-NFB	Post-NFB	Three-month follow-up	Pre-NFB	Post-NFB	Three-month follow-up
CAPS-total mean (SD)	36.52 (9.71)	23.19 (15.37)^a^	23.65 (13.71)^a^	39.94 (7.83)	32.78 (12.27)	31.78 (12.89)
% CAPS reduction		36.5% (clinically meaningful reduction)	35.2% (clinically meaningful reduction)		17.9%	20.4%
Result summary		Reduced compared to Pre-NFB (*P* = 0.003, *dz* = 0.77)	Reduced compared to Pre-NFB (*P* = 0.004, *dz* = 0.75)		*ns* (*dz* = 0.56)	*ns* (*dz* = 0.63)

a Indicates significantly reduced clinical measures within a PTSD group as compared to pre-NFB baseline, statistical significance corrected for multiple comparisons (*P* < 0.008).

Notably, 60.0% of participants in the experimental NFB group at the three-month follow-up assessment no longer met diagnostic criteria for PTSD (as compared to 33.3% of participants in the sham-control group). All PTSD patients meeting remission had clinically significant changes in CAPS corresponding to a greater than 30% reduction in PTSD severity scores.^67^ More specifically, for the experimental NFB group, the mean reduction in CAPS score was clinically significant^67^ when comparing baseline to post-NFB CAPS score (36.5%) and when comparing baseline to three-month follow-up CAPS scores (35.2%). Conversely, the mean change in CAPS scores in the sham-control group was below threshold when comparing baseline to post-NFB (17.9%) and when comparing baseline to three-month follow-up (20.4%) (see Table 2).

### EEG comparisons at baseline: PTSD versus neurotypical control group

Our main hypotheses and analyses were focused on testing for changes in the alpha band (8–12 Hz), based on previous studies showing abnormal alpha power in PTSD and the fact that the neurofeedback protocol selectively targeted this frequency. No significant differences were found in relative alpha power between the experimental NFB and sham-control groups at baseline, indicating a successful randomization in terms of this EEG phenotype.

**Figure 3:**
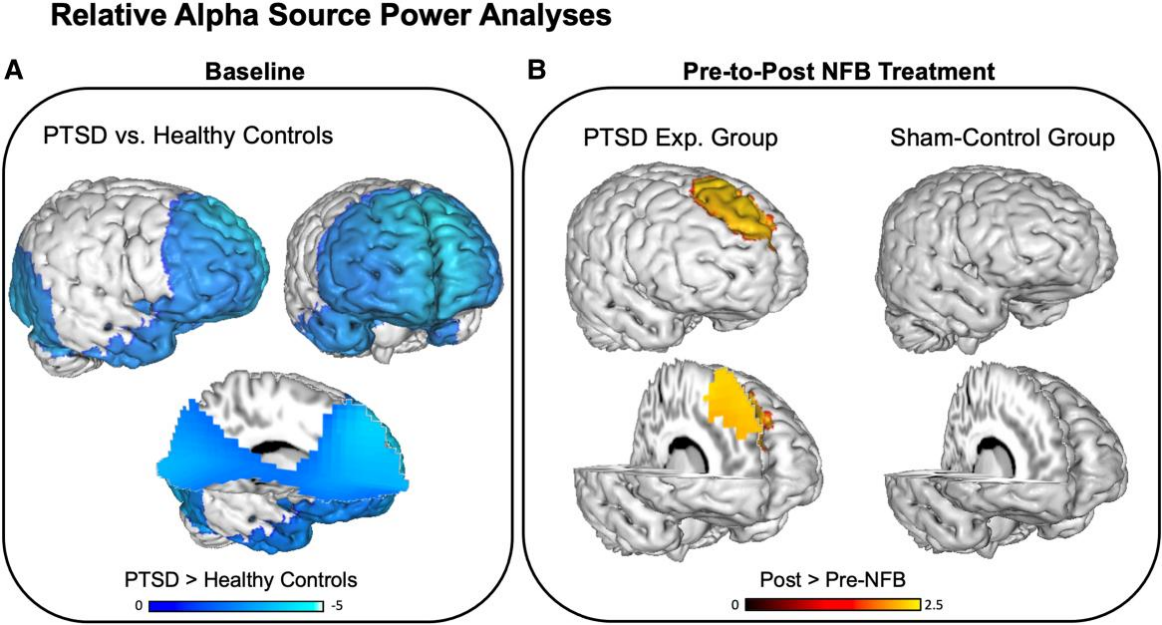
**Treatment induced changes in relative alpha source power.** (**A**) Reduced relative alpha source power at baseline among individuals with PTSD (pooled experimental NFB and sham-control group, *n* = 38) as compared to neurotypical healthy controls (*n* = 32), with minima in the medial frontal gyrus and the cuneus. (**B)** The ‘alpha rebound effect’ or alpha resynchronization in the experimental NFB group, corresponding to an increase in relative alpha power localized to the medial frontal gyrus, post as compared to pre-NFB intervention. No significant effect was detected for the sham-control group. Contrasts: PTSD > Healthy Controls = Relative alpha source power at baseline in the pooled PTSD group as compared to the neurotypical healthy control group, Post- > Pre-NFB = Relative alpha source power post as compared to pre-neurofeedback intervention. The blue bar indicates reduced relative alpha power, red/yellow bar indicates increased relative alpha power. Displayed results are *t*-values, and clusters are *P* < 0.05 FDR corrected.

As shown in Fig. 3A, at baseline individuals with PTSD (pooled experimental NFB and sham-control group, *n* = 38) displayed significantly reduced relative alpha power as compared to neurotypical healthy controls (*n* = 32), where maximum differences were focused within the medial frontal gyrus (BA8, BA9, BA10; *x* = −5, *y* = 52, *z* = 36; *T*_max_ = 4.34, *P* < 0.05 FDR corrected) and the cuneus (BA18; *x* = −4, *y* = −67, *z* = −1; *T*_max_ = 3.15, *P* < 0.05 FDR corrected).

Please see Supplementary Fig. 1 for statistical comparisons within other EEG bands (i.e. delta, theta and beta bands). In summary, at baseline individuals with PTSD demonstrated greater relative delta power in the medial frontal gyrus (BA8, BA9, BA10; *x* = −8, *y* = 52, *z* = 39; *T*_max_ = 3.34, *P* < 0.05 FDR corrected) and the cuneus (BA17, BA18; *x* = −26, *y* = −104, *z* = −15; *T*_max_ = 3.01, *P* < 0.05 FDR corrected) as compared to neurotypical healthy controls. In the theta band, individuals with PTSD exhibited greater relative power in the posterior cerebellum (*x* = 16, *y* = −80, *z* = −18; *T*_max_ = 2.66, *P* < 0.05 FDR corrected) and reduced relative power in the superior frontal gyrus (BA6, BA8; *x* = 4, *y* = 31, *z* = 60; *T*_max_ = 3.72, *P* < 0.05 FDR corrected). In the beta band, individuals with PTSD displayed greater power globally, but with maxima in the supplementary motor area (BA6; *x* = 16, *y* = −5, *z* = 72; *T*_max_ = 5.57, *P* < 0.05 FDR corrected).

### EEG comparisons pre- versus post-NFB: PTSD experimental and sham-control groups

As depicted in Fig. 3B, when comparing relative alpha power pre- versus post-intervention, the experimental NFB group demonstrated a significant increase in relative alpha power localized to the medial frontal gyrus (BA8, BA9, BA10; *x* = 7, *y* = 22, *z* = 48; *T*_max_ = 2.08, *P* < 0.05 FDR corrected). By contrast, as illustrated in Fig. 3B, we did not observe significant changes in relative alpha power for the sham-control group (both at *P* < 0.05 FDR corrected and uncorrected thresholds). However, the Group × Time interaction was not significant. We also did not detect significant correlations between pre-to-post changes on the CAPS and relative alpha power at this conservative FDR-corrected threshold.

Please see Supplementary Fig. 1 for pre- versus post-NFB statistical comparisons within other EEG bands (i.e. delta, theta and beta bands). In summary, the experimental NFB group demonstrated significant pre-to-post changes within the beta band, with relative power reductions in the right anterior cingulate and insula (BA13, BA33, BA47; *x* = 6, *y* = 9, *z* = 23; *T*_max_ = 2.18, *P* < 0.05 FDR corrected). On the other hand, the sham-control group displayed a significant reduction in delta relative power within the left precentral gyrus (BA4, BA6; *x* = −26, *y* = −17, *z* = 54; *T*_max_ = 3.01, *P* < 0.05 FDR corrected).

### Conjunction analysis: baseline alpha power abnormalities versus therapeutic changes in alpha power post-NFB

**Figure 4:**
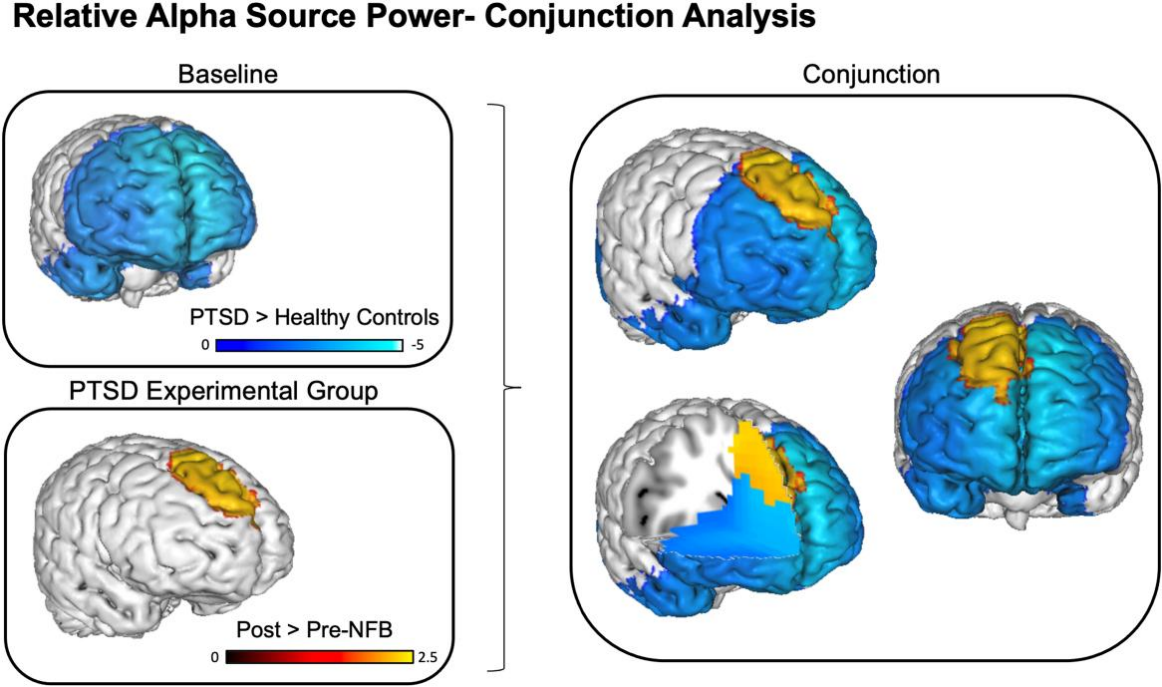
**Conjunction analysis for treatment induced changes in relative alpha source power.** *Top left*: baseline differences between the pooled PTSD group and the neurotypical healthy control group, corresponding to reduced relative alpha power. *Bottom left*: baseline reductions were partly normalized in the PTSD experimental group only, via alpha resynchronization pre-to-post NFB in the medial frontal gyrus. *Right*: illustration of the conjunction analysis which confirms the anatomical overlap with respect to baseline versus pre-to-post NFB tests. Contrasts: PTSD > Healthy Controls = Relative alpha source power at baseline in the pooled PTSD group as compared to the neurotypical control group, Post- > Pre-NFB = Relative alpha source power post as compared to pre-neurofeedback intervention. The blue bar indicates reduced relative alpha power, red/yellow bar indicates increased relative alpha power. Displayed results are *t*-values, and clusters are *P* < 0.05 FDR corrected.

Lastly, we tested whether regions displaying abnormally reduced relative alpha power among PTSD patients at baseline spatially overlapped with those that demonstrated a therapeutic increase in relative alpha power post-NFB. In other words, was there evidence for an anatomically-selective ‘normalization’ of alpha rhythms that were abnormal a priori? We addressed this question by utilizing a conjunction analysis, with the aim of identifying the overlapping or ‘in common’ voxels that were significant across two orthogonal contrasts: the group-wise baseline difference test (i.e. Fig. 3A) and the pre- versus post-NFB test (i.e. Fig. 3B). Here, a formal test for a conjunction (i.e. logical AND) requires that all the comparisons in the conjunction are individually significant at *P* < 0.05.^68^ As can be seen in Fig. 4, we identified a single overlapping cluster of alpha source power within the (mainly right) medial frontal gyrus (BA8, BA9, BA10). According to the Yeo 7-network functional parcellation, part of this cluster belongs to the anterior node of the default mode network.

### Neurofeedback performance and EEG spectral analysis

**Figure 5:**
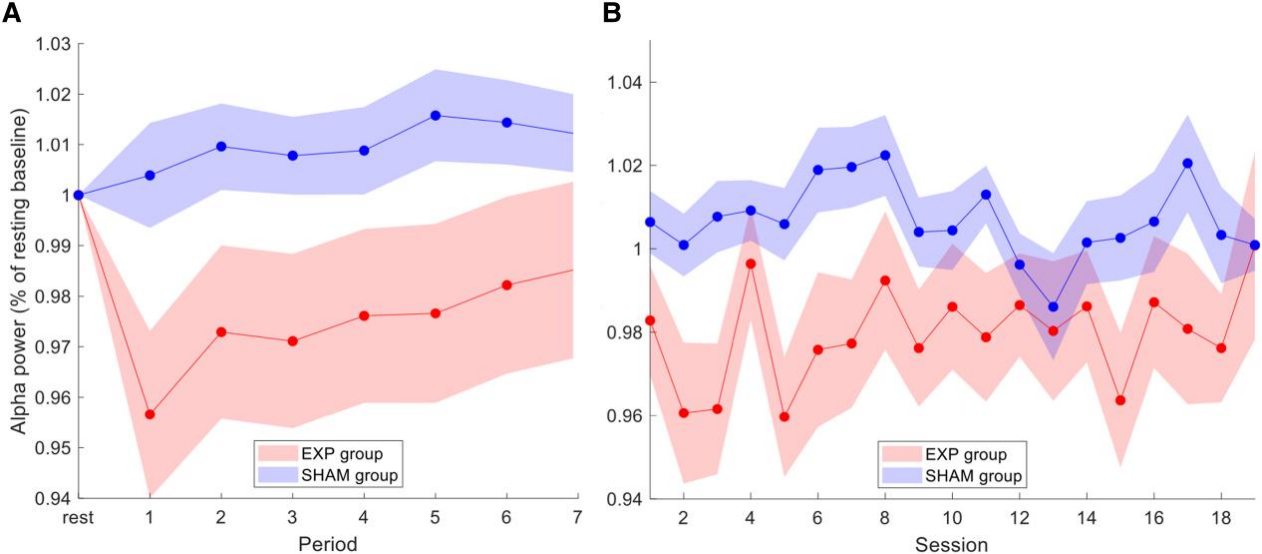
**Alpha power during neurofeedback training. (A**) *Within-session* alpha amplitude for the experimental NFB group and the sham-control NFB group averaged over all NFB training sessions (1–19). Rest represents the initial three-minute resting-state recording directly before training, where the subsequent feedback training was divided into seven periods (over 20 min total). Alpha amplitude at the feedback site (channel Pz) was expressed as % change relative to the rest baseline of the respective session. The alpha amplitude time course *within* each session differed significantly between the experimental and sham-control NFB groups [Group × Period interaction, *F*(7,252; Greenhouse–Geisser adjustment 2.44, 87.68) = 2.40, *η*_p_^2^= 0.062, *P* < 0.05 one-tailed (Greenhouse–Geisser corrected)]. (**B**) *Across-session* alpha amplitude for the experimental NFB group and the sham-control NFB groups averaged over all training periods (1–7). Alpha amplitude was expressed as % change relative to the rest baseline of the respective session. Our ANOVA revealed a significant main effect of group between experimental and sham-control NFB groups [Group, *F*(1,36) = 3.50, *P* < 0.05 one-tailed], as well as a significant main effect of session [Session, *F*(18,648; Greenhouse–Geisser adjustment 9.1, 328.20) = 2.03, *η*_p_^2^=0.054, *P* < 0.05 (Greenhouse–Geisser corrected)]. Acronyms: Exp = experimental neurofeedback PTSD group, Sham = sham-control PTSD group. Shaded areas indicate ± 1 SEM.

In order to confirm that NFB resulted in differential changes of the controlled parameter (alpha amplitude), we examined alpha power during the 20-minute NFB sessions (6 × 3-minute training periods, and 1 × 2-minute training period) in both the experimental and sham-control NFB groups. Training alpha power (within periods 1–7) was defined as the average percent change from baseline alpha power within each respective session (i.e. the initial rest period of that session). As depicted in Fig. 5A, and consistent with the NFB protocol, the experimental NFB group exhibited a more sustained reduction of alpha amplitudes as compared to the sham-control NFB group. For the feedback channel Pz, the alpha amplitude time course *within* each session differed significantly between the experimental and sham-control NFB groups [Group × Period interaction, *F*(7,252; Greenhouse–Geisser adjustment 2.44, 87.68) = 2.40, *η*_p_^2^= 0.062, *P* < 0.05 one-tailed (Greenhouse–Geisser corrected)]. Counterintuitively, as shown in Fig. 5A, the NFB group displays a return to baseline values evidenced by gradually rising values of relative alpha power from the third period onwards. We interpret this as a potential homeostatic ‘rebound’ response that may already be triggered during the neurofeedback session. Longitudinal changes in alpha percent change *across* all training sessions are depicted in Fig. 5B. Here, our ANOVA revealed a significant main effect of group between experimental and sham-control NFB groups [Group, *F*(1,36) = 3.50, *P* < 0.05 one-tailed], and a significant main effect of session [Session, *F*(18,648; Greenhouse–Geisser adjustment 9.1, 328.20) = 2.03, *η*_p_^2^=0.054, *P* < 0.05 (Greenhouse–Geisser corrected)]. The Group × Session interaction was non-significant. The main effect of Group indicates the experimental NFB group demonstrated lower percent alpha power values compared to the sham-control group, on average, over the whole course of the treatment. Interestingly, most participants reported that they tried to ‘quiet their mind’ by decreasing mind-wandering thoughts and increasing visual attention to the NFB screen as a means to gain control over their feedback signal.

## Discussion

In this RCT among individuals with PTSD, we report significant reductions on PTSD severity scores and increased rates of PTSD remission in the experimental group following 20 sessions of NFB training. Importantly, this is the first double-blind RCT in PTSD to concurrently track aberrant PTSD brain rhythms before and after NFB treatment.

Overall, we provide evidence for a pre-to-post normalization of alpha rhythmicity via NFB training (alpha rebound), which was restricted to the experimental NFB group and was not observed in the sham-control group. As compared to neurotypical healthy controls at baseline, PTSD patients demonstrated significantly decreased relative alpha power predominantly within the anterior portion of the DMN (medial frontal gyrus). After the 20-week EEG-NFB intervention, the experimental NFB group demonstrated a resynchronization (rebound) of alpha power mainly over right dorsomedial prefrontal cortex (dmPFC) areas of the DMN, an effect that was not observed for the sham-control group. Conjunction analyses confirmed the spatial overlap of aberrant alpha power at baseline and regions which demonstrated a therapeutic alpha resynchronization post-NFB, indicating a normalization of the neural circuitry traditionally implicated in PTSD.^26,31,37-41^

Clinically, at the three-month follow-up assessment, 60.0% of participants in the experimental NFB group no longer met diagnostic criteria for PTSD, a remission rate which was significantly higher than the sham-control group (33.3%). Here, only average reductions of CAPS scores in the experimental NFB group were clinically significant (i.e. > 30% reduction^67^) when comparing baseline to post-NFB CAPS scores (36.5%) and when comparing baseline to three-month follow-up CAPS (35.2%). Conversely, the average change in CAPS scores in the sham-control group was not significant when comparing baseline to post-NFB (17.9%) and when comparing baseline to three-month follow-up (20.4%). It is important to note here that the experimental group remission rate of 60.0% is comparable to those of current, gold standard treatments for PTSD.^6,19,21,69^ Additionally, we experienced no patient dropouts from the NFB treatment, which speaks to the tolerability, feasibility and safety of alpha NFB among individuals with PTSD.

### Reduced baseline alpha power within the default mode network in PTSD

In the current study, we replicated previous findings reporting attenuated resting-state alpha rhythms at baseline among individuals with PTSD, as compared to neurotypical healthy controls.^31,36,47,70^ Aberrant alpha rhythms and associated DMN connectivity are both highly implicated in the pathophysiology of PTSD.^31,71^ In a recent machine learning study, alpha oscillations have been shown to be a significant longitudinal predictor of PTSD symptoms.^72^ Furthermore, it has been suggested that hypoactive resting-state alpha is associated with disrupted E/I balance and DMN functioning^71^ at rest in PTSD.^31,47,52,70^ Indeed, using high-density EEG source analyses, alpha-rhythm reductions have been observed previously within the main hubs of the DMN (dmPFC and PCC).^31^ In PTSD, functional disruptions within the DMN are hypothesized to underscore negative self-referential thoughts as well as altered social cognition, bodily self-consciousness and autobiographical memory.^71,73-79^ Several fMRI studies suggest that connectivity within the posterior community of the DMN (PCC and precuneus) may be intact or exacerbated relative to decreased connectivity within the anterior community of the DMN (dmPFC).^49,80-82^ The DMN is also characterized by less overall efficiency of communication across the network with increased segregation among individuals with PTSD when examining graph theoretical analyses with fMRI.^49^ Additionally, studies exploring fMRI seed-based functional connectivity patterns within the DMN at rest have revealed decreased coupling between the PCC, vmPFC and other DMN structures, which together have been associated with PTSD symptoms.^71,74-86^

Notably, decreased resting-state alpha has also been hypothesized to mediate clinical symptoms of chronic hyperarousal related to altered functioning within the SN.^26,28,31,37-41^ Specifically, increased activity/connectivity within the SN has been shown to be associated with PTSD symptoms of hyperarousal, hypervigilance, avoidance and altered interoception.^9,11,77,87-89^ In support of this, in the current RCT, we found disrupted DMN connectivity and hyperconnectivity of the SN at baseline in the PTSD group as compared to neurotypical controls, evaluated using resting-state fMRI data and reported elsewhere.^11^ Importantly, arousal is also a key dimension of the RDoC,^90^ and is thus a critical axis for understanding and treating PTSD as well as anxiety disorders more generally.^24^

### Neurofeedback restores alpha power within the default mode network in PTSD

Post-NFB intervention, we observed restored relative alpha power in the experimental NFB group only. Here, conjunction analyses confirmed that this alpha resynchronization or ‘alpha rebound effect,’ occurred within the same regions displaying reduced alpha power among PTSD patients at baseline. In line with this, we have found previously that a single-session of NFB normalized (increased) alpha rhythms among PTSD patients post-intervention towards levels found in neurotypical healthy controls.^47^ Our results now provide long-term evidence that by homeostatically restoring cortical inhibition and E/I balance in PTSD, alpha resynchronization may indeed be a key neural mechanism underlying the therapeutic benefits of NFB.^24^ Interestingly, we also found significant reductions in relative beta power post-intervention within the experimental NFB group, where elevated beta rhythms have been consistently reported as a biomarker of PTSD psychopathology^36^ and were similarly detected at baseline in the current study.

### Homeostatic plasticity of the alpha rhythm and cortical E/I balance

Using transcranial magnetic stimulation, we have previously reported that alpha power modulations induced by NFB were negatively correlated with changes in cortical excitability and disinhibition.^91^ Crucially, the ‘alpha rebound effect’ that was observed in the current study replicates previous findings from both single-session^18,47^ and multi-session NFB studies^46^ and has been proposed to occur via homeostatic neuroplasticity mechanisms.^18,37,41,47^ Homeostatic neuroplasticity refers to the brain’s *self-tuning capacity* to regulate E/I balance^91,92^ and has been observed in NFB studies as a counterintuitive shift in alpha oscillations in the opposite direction of training.^18,41,47,91^ In other words, this corresponds to training neural oscillations towards one extreme, and observing a rebound of their oscillatory activity in the opposite direction, such that pathological brain rhythms become normalized.^92^ In this study, we found evidence to suggest that this rebound of inhibitory alpha rhythms may already be triggered partway through the NFB session. Indeed, alpha desynchronizing NFB protocols may be a fruitful avenue by which to normalize resting-state alpha rhythms that are known to be decreased in PTSD and highly associated with trauma-related symptoms.^18,46,47^ Behaviourally, we have found previously that alpha rebound post-NFB correlated with reduced hyperarousal symptoms among individuals with PTSD, as well as normalized resting-state connectivity patterns within the DMN and SN.^18,47,52^ Elsewhere, alpha rebound following seven sessions of alpha desynchronizing NFB has also been shown to lead to clinically relevant reductions in symptoms among individuals with PTSD using low-cost wearable EEG-based neurotechnology.^46^ Taken together, this homeostatic return of alpha power and E/I balance may be a critical neurobiological mechanism underlying the clinical benefits of NFB in PTSD.^18,37,47,52^

In the current RCT, we observed alpha resynchronization specifically within the dmPFC in the experimental NFB group, which is the main hub of the anterior DMN.^93^ This finding provides converging evidence with our resting-state fMRI analysis on the same RCT dataset, which revealed increased anterior DMN connectivity to the same dmPFC region as a function of NFB.^11^ Collectively, these findings suggest restored engagement of anterior nodes in the DMN that are typically disengaged/segregated in PTSD.^11,49,80,82^ Similarly, these findings are also supported by our previous single-session EEG-NFB experiments investigating alpha downregulation in PTSD, where post-training we found that increased DMN connectivity with the dmPFC was associated with reduced hyperarousal symptoms.^18^ Additionally, a recent study by Popescu and colleagues^94^ found that dysregulated alpha oscillations in the middle frontal gyrus was associated with PTSD severity scores and performance during a working memory task. Taken together, these results suggest that normalizing dmPFC functioning may be involved in restoring anterior nodes of the DMN, and that the dmPFC is a critical area associated with alpha resynchronization and therapeutic reductions in PTSD symptoms.

### Limitations and future directions

The current study was not pre-registered as a clinical trial as ethical (REB) approval occurred before this became a standard practice in the field. As such, we were highly restrictive with the clinical outcome measures we examined, and all mechanistic hypotheses were strictly based on previously published data. Additionally, significant group differences on CAPS scores were not found when comparing PTSD experimental and sham-control groups directly, either immediately post-NFB or at the three-month follow-up. Given that trending reductions on PTSD scores were also observed in the sham-control group, we speculate that this may be due to participants in both groups building supportive relationships with trauma-informed clinicians during regular psychoeducation visits to the clinic. During these sessions, all participants were encouraged to be ‘mindfully’ present and grounded for 20 min once a week during the NFB trial. Hence, future studies designed to compare mental strategies (for example mindfulness) and no-training control groups (for example waitlist and treatment as usual control groups)^59^ with ideally powered larger sample sizes are warranted. Furthermore, we did not detect a direct association between changes in CAPS scores and changes in alpha power from baseline, where ideally powered future investigations may be required to identify such associations. Future studies might also aim to directly compare NFB protocols that up- or down-regulate different EEG oscillations (i.e. delta, theta, alpha or beta), and examine different electrode placements and NFB training schedules. Moreover, the impact of medication, prior psychological therapies (including sequencing/layer treatments), cumulative trauma and duration of symptoms should be investigated in larger sample sizes, especially in the context of treatment-resistant PTSD. Finally, although we administered identical levels of reward during NFB and group blinding was maintained throughout the trial, a potential disadvantage of yoked signal feedback may be a lack of signal controllability experienced by participants.^59^

## Conclusions

In this 20-session, double-blind, randomized controlled trial of alpha-rhythm EEG-NFB among individuals with PTSD, we observed therapeutic changes in aberrant PTSD brain rhythms as a function of NFB. Our central goal was to provide mechanistic evidence underlying clinical improvements by examining change in relative alpha power over treatment. In line with the current knowledge base, we observed significantly reduced relative alpha power at baseline in the PTSD as compared to the neurotypical healthy control group, primarily within areas of the anterior default mode network (medial frontal gyrus). We found that only PTSD patients in the experimental NFB group demonstrated alpha resynchronization (alpha rebound) within areas that previously displayed reduced alpha power at baseline. Further, we found significantly decreased PTSD severity scores in the experimental NFB group only, when comparing post-NFB and three-month follow-up scores to baseline measures, with clinically significant remission rates (60.0%). Our results suggest that alpha desynchronizing NFB has the capacity to increase/normalize resting-state alpha rhythms that are known to be decreased in PTSD and highly associated with PTSD symptomatology. This study replicates previous observations reporting alpha-rhythm resynchronization following alpha desynchronizing NFB treatment. The current findings also suggest that the alpha rebound effect may be a critical neuroplastic mechanism underlying the therapeutic benefits of alpha-based NFB.

## Supplementary Material

fcad068_Supplementary_Data

## Data Availability

The data that support the findings of this study are available from the corresponding author upon reasonable request.

## Abbreviations

BEM =: boundary element method
CAPS =: clinician-administered post-traumatic stress disorder scale
CTQ =: childhood trauma questionnaire
DMN =: default mode network
dmPFC =: dorsomedial prefrontal cortex
E/I =: excitatory/inhibitory
FFT =: fast Fourier transformation
fMRI =: functional magnetic resonance imaging
MDD =: major depressive disorder
MDI =: multiscale dissociation inventory
mPFC =: medial prefrontal cortex
NFB =: neurofeedback
PCC =: posterior cingulate cortex
PTSD =: post-traumatic stress disorder
RCT =: randomized controlled trial
RDoC =: research domain criteria
REB =: research ethics board
SCID =: structured clinical interview for DSM
sLORETA =: standardized low-resolution electromagnetic tomography analysis
SN =: salience network
SPSS =: statistical package for the social sciences
vmPFC =: ventromedial prefrontal cortex
