# Supplementary material for: Homeostatic normalization of alpha brain rhythms within the default-mode network and reduced symptoms in post-traumatic stress disorder following a randomized controlled trial of electroencephalogram neurofeedback
Source: Brain Commun. 2023 Mar 16;5(2):fcad068. doi: 10.1093/braincomms/fcad068 (PMC10090479; doi:10.1093/braincomms/fcad068)
Supplement: fcad068_Supplementary_Data [file fcad068_supplementary_data.pdf]

Supplementary Results

EEG Comparisons at Baseline: PTSD vs. Neurotypical Control Group

At baseline, individuals with PTSD demonstrated greater relative delta power in the medial frontal gyrus (BA8, BA9, BA10; x = -8, y = 52, z = 39; Tmax = 3.34, $p < 0.05$ FDR corrected) and the cuneus (BA17, BA18; x = -26, y = -104, z = -15; Tmax = 3.01, $p < 0.05$ FDR corrected) as compared to neurotypical healthy controls. In the theta band, individuals with PTSD exhibited greater relative power in the posterior cerebellum (x = 16, y = -80, z = -18; Tmax = 2.66, $p < 0.05$ FDR corrected) and reduced relative power in the superior frontal gyrus (BA6, BA8; x = 4, y = 31, z = 60; Tmax = 3.72, $p < 0.05$ FDR corrected). In the beta band, individuals with PTSD displayed greater power globally, but with maxima in the supplementary motor area (BA6; x = 16, y = -5, z = 72; Tmax = 5.57, $p < 0.05$ FDR corrected).

EEG Comparisons Pre vs. Post-NFB: PTSD Experimental and Sham Control Groups

The experimental NFB group demonstrated significant pre-to-post changes within the beta band, with relative power reductions in the right anterior cingulate and insula (BA13, BA33, BA47; x = 6, y = 9, z = 23; Tmax = 2.18, $p < 0.05$ FDR corrected). On the other hand, the sham control group displayed a significant reduction in delta relative power within the left precentral gyrus (BA4, BA6; x = -26, y = -17, z = 54; Tmax = 3.01 $p < 0.05$ FDR corrected).

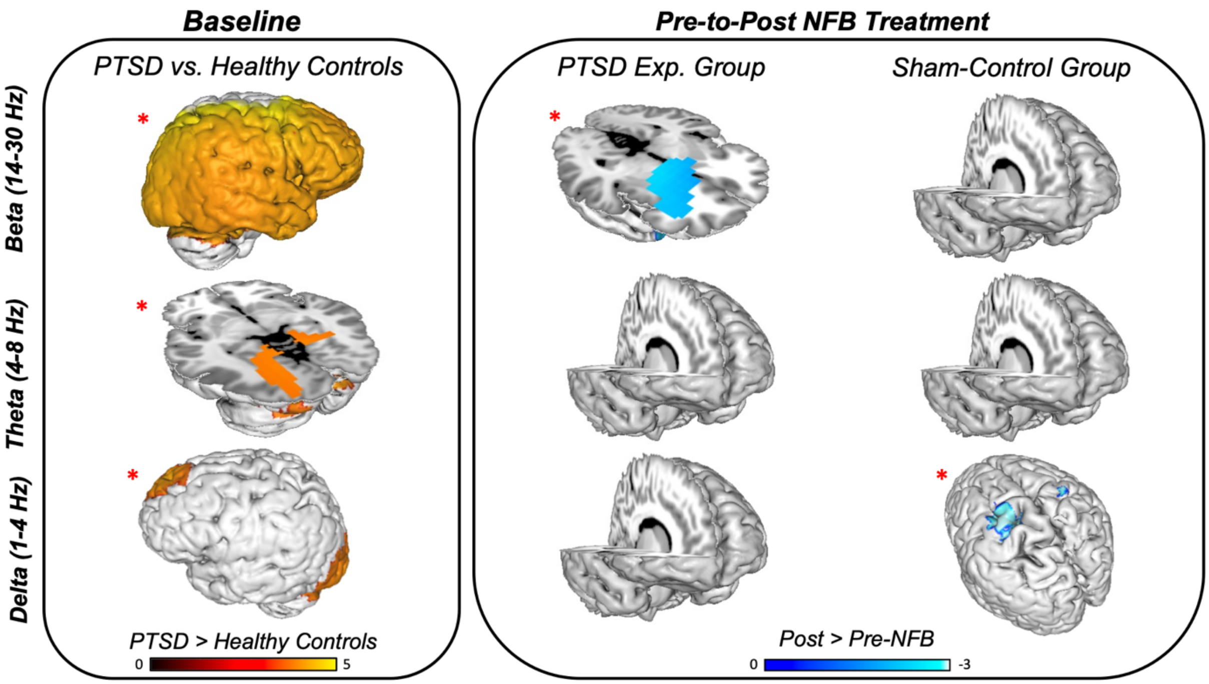

Supplementary Figure 1: Relative power comparisons in other EEG bands. Left panel: PTSD > Healthy Controls= Relative EEG source power at baseline in the pooled PTSD group as compared to the neurotypical healthy control group. Right Panel: Post > Pre Treatment= Relative source power post as compared to pre neurofeedback/sham intervention. Blue colours indicate reduced relative power, red/yellow colours indicate increased relative power. NFB= Neurofeedback. Displayed results are t-values, and clusters are $p < 0.05$ FDR corrected.
